# Patient participation in patients with heart failure receiving structured home care - a prospective longitudinal study

**DOI:** 10.1186/s12913-014-0633-y

**Published:** 2014-12-18

**Authors:** Lena Näsström, Tiny Jaarsma, Ewa Idvall, Kristofer Årestedt, Anna Strömberg

**Affiliations:** Department of Medical and Health Sciences, Linköping University, Linköping, 581 85 Sweden; Department of Social and Welfare studies, Linköping University, Norrköping, Sweden; Department of Care Science, Malmö University, and Department of Intensive Care and Perioperative Medicine, Skåne University Hospital, Malmö, Sweden; School of Health and Caring Sciences, Linnaeus University, Kalmar, and Palliative Research Centre, Ersta Sköndal University Collage and Ersta Hospital, Stockholm, Sweden; Department of Medical and Health Sciences, Linköping University, and Department of Cardiology, County Council of Östergötland, Linköping, Sweden; Department of Medical and Health Sciences, Linköping University, Linköping, Sweden

**Keywords:** Heart failure, Home care services, Multi-disciplinary care, Patient education, Patient involvement, Patient participation, Self-care, Social support

## Abstract

**Background:**

Patient participation is important for improving outcomes, respect for self-determination and legal aspects in care. However, how patients with heart failure view participation and which factors may be associated with participation is not known. The aim of this study was therefore to describe the influence of structured home care on patient participation over time in patients diagnosed with heart failure, and to explore factors associated with participation in care.

**Methods:**

The study had a prospective pre-post longitudinal design evaluating the influence of structured home care on participation in patients at four different home care units. Patient participation was measured using 3 scales and 1 single item. Self-care behavior, knowledge, symptoms of depression, socio- demographic and clinical characteristics were measured to explore factors associated with patient participation. Repeated measure ANOVA was used to describe change over time, and stepwise regression analyses were used to explore factors associated with patient participation.

**Results:**

One hundred patients receiving structured heart failure home care were included. Mean age was 82 years, 38 were women and 80 were in New York Heart Association functional class III. One aspect of participation, received information, showed a significant change over time and had increased at both six and twelve months. Better self-care behavior was associated with all four scales measuring different aspects of participation. Experiencing lower degree of symptoms of depression, having better knowledge, being of male sex, being of lower age, cohabiting and having home help services were associated with one or two of the four scales measuring different aspects of participation.

**Conclusion:**

Patients experienced a fairly high level of satisfaction with participation in care at baseline, and there was a significant improvement over time for participation with regard to received information after being admitted to structured home care. Higher level of patient participation was consistently associated with better self-care behavior. This study shows that patient participation may need to be further focused upon, and that the association with self-care may be interesting to target in future interventions.

## Background

Participation in care is important in order to improve outcomes for patients [1]. It is also vital when it comes to respecting a person’s right to self-determination and meeting legal aspects of care [2,3]. It is particularly important to promote and facilitate participation in care among patients suffering from chronic illness, such as heart failure (HF) [4,5]. Heart failure is a common condition, and in developed countries the estimated prevalence of symptomatic HF is 1-2% of the population [6,7]. Heart failure prevalence increases with age and is one of the main causes for hospitalization among people aged 65 years or older, with a substantial risk for re-hospitalizations, leading to high medical costs [7]. The organization of HF care has seen many changes in the previous decades, illustrated by the introduction of HF management programs. There has also been a development from inpatient to outpatient care, and care is also to a greater extent provided in the patients’ homes [8,9]. Previous studies with structured, nurse-led HF home care, have had a positive impact with significantly decreased re-admissions to hospital [10,11], as well as increased survival [11] when compared to follow-up in primary care. However, a more recent study comparing nurse-led care in a home-based setting to a clinic-based setting showed no significant differences between the groups regarding unplanned hospitalizations or death during the 12–18 month follow-up [12]. A considerable part of HF care is already provided in the patients’ homes, and from the patient’s view home care can be an opportunity to live a more independent life and avoid hospitalizations [13].

### Patient participation

According to the International Classification of Functioning, Disability and Health, participation is “a person’s involvement in a life situation”, where there is an interaction between activity, participation, health conditions, body functions and structures, and environmental and personal factors [14]. When the concept was analyzed, the findings revealed that participation includes an existing relationship, shared information and knowledge, a will to move power and control to the patient, and mutual activity in intellectual and/or physical activity [1,15]. This mutual activity is expected to have positive associations. The concepts of patient participation and involvement can either be synonymous, or to some extent have different meanings [15]. In this paper the concepts are used synonymously. Patient participation includes having knowledge, interaction with health care professionals [16] and decision-making, although the preferences for this differ [17-19]. Patients diagnosed with HF have described participation as having confidence, understanding, seeking and receiving a sense of control [20], or as communication with health care professionals (HCP), accessibility to care, active involvement in care, trustful relations with HCP’s and options for decision-making [21]. These findings illustrate that there is no uniform definition of patient participation, and that the concept is complex and includes several different aspects. In addition to previous studies, interventions to increase patient participation have shown that treatment outcomes in diabetes control [22,23], physical activity [24], and adherence to drug treatment in depression [25] improve significantly. Based on previous research, we can assume that there may be a link between patient participation and self-care, which could be expected to result in improved health outcomes, but other factors probably also influence these outcomes.

### Factors associated with patient participation

Since there is an association between the patient’s ability to self-care and management of various aspects of their condition, improving HF self-care is fundamental for improving outcomes [26-28]. By engaging in self-care, patients become active participants [4], and self-care is described as a cognitive process initiated by the patient to maintain health and manage illness and disease [29]. Different aspects of decision-making are involved in self-care, and these are influenced by interactions between personal characteristics, the problem, and environmental factors [30]. Knowledge is a foundation for HF self-care [31], and being able to apply knowledge about one’s condition contributes to patient participation [20]. Patients might find it difficult to understand their symptoms and make a connection to deteriorating HF [32,33], and adhere to a HF regimen [34]. Furthermore, due to typical symptoms and limitations, living with HF influences a person’s life, often decreasing the patient’s quality of life and reducing functional capacity [7]. Depressive symptoms is also frequent among patients with HF, and up to one third of patients with HF have depressive symptoms [35,36]. Patients’ preferences for participation could also be influenced by different socio-demographic factors. Studies from other fields have shown that preferences for a higher degree of participation in decision-making are associated with e.g. female sex [37-39], lower age [37,39], higher level of education [38,39], and living alone [38]. On the other hand, other studies have not been able to identify any differences related to sex [40,41] or age [41]. Furthermore, in a review, Hubbard and co-workers [42] state that it has not yet been clarified how socio-demographic factors influence patient participation.

### Rationale for the study

Earlier research indicates that patient participation is of importance, but little is still known of how patients diagnosed with HF view participation, what factors are related to patient participation, how patient participation is influenced in specific care settings, and if there is a change in participation over time. In order to understand and improve HF care, it may be of importance to identify a possible association between patient participation, self-care and HF knowledge. We also assume that depressive symptoms may affect patients’ ability to participate, as participation includes different aspects of activity, as well as belief in your own ability. However, little is known of how symptoms of depression in patients with HF are associated with patient participation. A better understanding whether socio-demographic factors are associated with patient participation among patients with HF can be useful for improving care. These aspects are of importance in order to improve health outcomes, care, and patient satisfaction with care.

### Aim

The aim of this study was to describe the influence of structured home care on patient participation over time in patients diagnosed with HF, and to explore factors associated with patient participation in care.

## Methods

### Design, participants and settings

The study had a prospective pre-post longitudinal design, evaluating the influence of structured home care on patient participation, with a 12-month follow-up period. Inclusion criteria were patients 18 years or older, and diagnosed with HF as defined by the European Society of Cardiology [7]. Exclusion criteria were expected survival less than three months, cognitive impairment or mental illness that could affect informed consent or active participation, and difficulties to speak or understand Swedish. A consecutive sample of Swedish patients diagnosed with HF was used. All eligible patients were assessed for study participation at four home care units in Sweden during February 2010 and October 2011. Patients were approached by a study nurse and received both verbal and written information about the study. Two units were situated in a metropolitan area and two in a medium-sized city (about 145 000 inhabitants). These units offered home care by a team consisting of a minimum of nurses and physicians, and patients could contact the team at all hours. The team members were specialists in general care as well as being trained in HF and structured home care according to *The heart failure at home model* [43]. In all units, the HCPs were introduced to the home care model during an educational day at each of the different units, where the components of the model were thoroughly explained. There was also a discussion on how the model could be implemented in clinical practice. *The Heart Failure at Home Model* consists of six components for home-based management of HF: 1) A multidisciplinary team, minimum physicians and nurses 2) Competency-based staff education 3) Joint care plans and/or care paths 4) Optimized treatment according to guidelines 5) Educational strategies for patients/families/caregivers, and 6) Increased accessibility to care. The model aims to facilitate patient care and focuses on values such as safety, participation and having knowledge about the illness and treatment. Nurses at each unit received additional education in *The heart failure at home mode.* These nurses were responsible for supporting their colleagues in the implementation of the model. Furthermore, the research team continuously reinforced and followed the process. Through chart reviews and audits, e.g., monitoring of the care plans, access to care and educational strategies, the researchers ensured that the home care model was implemented throughout the study. Structured home care, based on the *Heart Failure at Home Model,* was given once the patient had completed the baseline questionnaire. All patients received all components of the intervention. The interval of patient contacts (home visits and telephone follow-up) was individualized based on the patient’s medical condition and educational needs.

### Data collection procedure

Data were collected at baseline and after 1, 6 and 12 months from patients’ self-reports gathered in a questionnaire and from medical records. The questionnaire included demographic questions and a battery of validated instruments to assess participation, self-care behavior, knowledge of HF and symptoms of depression.

### Assessment

#### Socio-demographic and clinical characteristics

Data on age, sex, education level, smoking habits and alcohol consumption were collected from patients’ self-reports. Data on cohabitation, housing, home help services, New York Heart Association (NYHA) functional classification, hospitalization, mortality, HF medication, blood pressure, pulse rhythm and classification of co-morbidities according to the Charlson Co-morbidity Index [44] were collected from medical records. The Charlson Co-morbidity index assign weighted from 1–6 for the presence of specific co-morbidities, with a possible range from 0–34.

#### Participation

To assess aspects of patient views on participation in care, a Swedish questionnaire developed by Arnetz and co-workers was used. The instrument has demonstrated good validity and reliability [45]. In this study, three of the instrument’s six scales and one single item were used. These items were selected in collaboration with the instrument developer and were considered applicable to patients with HF in a home care context. The first scale, *Patient involvement,* included six items of how patients define involvement, with a total score of 6–24. The second scale, *Information,* included five items on received information and explanations regarding medical condition, its course and treatment, with a total score of 5–20. The third scale, *Patient needs,* included seven items of how needs were fulfilled in terms of asking questions, understanding information and being treated with respect by health care professionals (HCP), with a total score of 7–28. All these items were rated on a four-point Likert-type scale, ranging from don’t agree at all (scored 1) to agree completely (scored 4), or from no, not at all (scored 1) to yes, to a great degree (scored 4). Higher scores indicate a more positive rating. Finally, a single item on overall satisfaction with involvement in care was graded on a numeric rating scale, ranging from 1 (not at all satisfied) to 10 (very satisfied). Since the instrument was developed for patients with myocardial infarction (MI) in hospital settings, the wordings in three items were changed slightly in order to adapt them to patients with HF in outpatient care. Cronbach’s α values in the three scales used in this study ranged between 0.80-0.88.

#### Self-care behavior

The European Heart Failure Self-care Behavior scale (EHFScB-9) consists of nine statements regarding self-care in HF and is tested for good validity and reliability [46]. Self-care behavior was estimated on a five-point Likert-type scale, ranging from 1 (completely agree) to 5 (completely disagree). The total score ranged from 9–45. Lower scores indicate better self-care behavior. Cronbach’s α was 0.72 in the present study.

#### Knowledge

The Dutch Heart Failure Knowledge Scale [47] consists of 15 multiple choice questions about knowledge of HF in general (4 items), HF treatment (6 items) and symptom/symptom recognition (5 items). A score of one was given for each correct answer and zero was given for incorrect answers. The total score ranged from 0–15. van der Wal and co-workers [47] found the instrument valid and reliable. A translation of the instrument into Swedish has been made, using both forward- and backward translation. To test the instrument’s internal consistency in this study, a Kuder Richardson coefficient (KR-20) was calculated (0.54).

#### Symptoms of depression

The Patient Health Questionnaire (PHQ-9) [48] with nine items measuring depressive symptoms during the last two weeks was used. PHQ9 has shown to be valid and reliable [49], and has also been validated in patients diagnosed with HF [50]. Each item was rated on a four-graded scale from not at all to nearly every day, scored from 0–3 with a total score of 0–27 points. In the present study, Cronbach’s α was 0.80.

### Data analysis

Descriptive statistics were used to describe the sample and study variables. To make the four scales (patient involvement, information, patient needs and overall satisfaction with involvement in care) comparable, values were calculated by converting the sum to a percentage of the maximum possible score (100%). This calculation was inspired by the instrument developer [45].

To describe the patients’ views of participation at the four measurement points that took place over a 12-month period, a repeated measure ANOVA was performed with the four scales patient involvement, information, patient needs and overall satisfaction with involvement in care [45]. Mauchly’s test of sphericity was used to evaluate if the variance in the differences between all possible pairs of groups (i.e., time) were equal. If this assumption was violated, the Huynh-Feldt correction was applied [51].

As a first step to explore factors associated with patients’ views of participation, a bivariate correlational analysis was performed, using Spearman’s rho correlation coefficient. This analysis was conducted at the first assessment. Variables were selected based on prior empirical evidence or theoretical assumptions hypothesized to be associated with patient participation. In the correlation analysis, the four scales patient involvement, information, patient needs and overall satisfaction were used. Furthermore, symptoms of depression (PHQ-9), self-care behavior (European Heart Failure Self-Care Behavior Scale), knowledge (Dutch Heart Failure Knowledge Scale), sex, age, cohabitation, housing, home-help services, education level, co-morbidities and NYHA class were included. Based on the results of the bivariate correlation, variables that were to be used as predictors in the regression analysis were determined if the p-value was <0.10. In a second step, stepwise linear regression analyses with backward elimination were performed. In these analyses, the four scales patient involvement, information, patient needs and overall satisfaction with involvement in care were used as outcome variables. Based on the findings from the correlation analysis, socio-demographics (age, sex, home-help service and cohabitation), clinical characteristics (NYHA class), knowledge (Dutch Heart Failure Knowledge Scale), symptoms of depression (PHQ-9), and self-care behavior (European Heart Failure Self-Care Behavior Scale), were included to determine predictor variables associated with participation. No variables indicated problems with multicolinarity, the variance inflation factor (VIF) was < 2 in all models of the regression analysis [52]. Except for the Bonferroni corrected post hoc tests, the significance level was set to < 0.05. Data were analyzed using IBM SPSS Statistics 20.

### Ethical considerations

The study followed the Principles of the Declaration of Helsinki, and participants gave written informed consent. Permission was granted by the Regional Ethical Review Board in Linköping (M210-09).

## Results

### Sample and descriptive information

**Figure 1:**
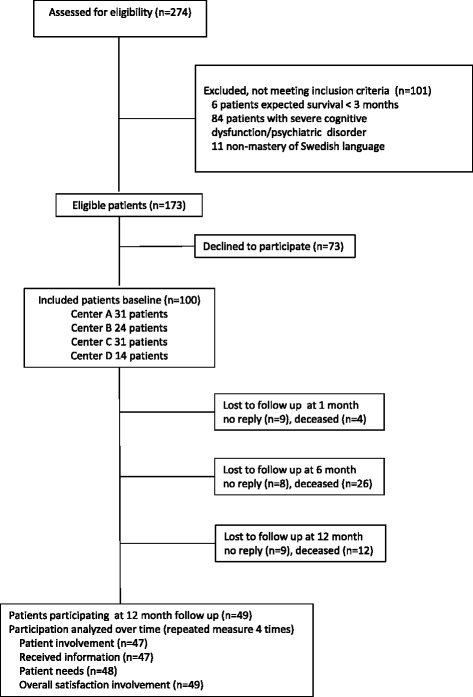
**Flow chart of the participants in the study.** Study enrollment, follow-up and analysis of different views of participation over time.

During the inclusion period 274 patients were eligible, resulting in 100 consecutively included patients at baseline, with 49 patients remaining at the fourth data collection point 12 months later (Figure 1). There were 62 men and the mean age was 82 years. Eighty of the participants were classified in NYHA class III and had a high prevalence of co-morbidities (4.0 ± 2.2) (Table 1). During the study period, 55 of 100 patients were hospitalized at some point and 42 died. Eighty-seven were prescribed beta-blockers, 95 loop-diuretics and 72 angiotensin –converting enzyme inhibitor/angiotensin receptor blockers. There were no significant differences regarding age and sex between the included patients and those eligible who did not want to participate.

**Table 1:** **Socio-demographic and clinical characteristics of patients at baseline (n = 100)**

**Socio-demographic and clinical characteristics**	
**Age** mean (SD)	81.7 (8.8)
**Male** n	62
**Cohabitation** n	52
**Housing** n	
Apartment	80
Own house	17
Block of service flats	3
**Home-help service** n	46
**Education level** n	
Elementary, primary and secondary school	67
High/trade school 2 years	5
High-school 3–4 years	10
Higher education/university	18
**Smoking** n	
Never smoked	37
Stopped smoking > 1 year ago	53
Stopped smoking > 1 month - < 1 year ago	2
Smoke regularly	8
**Alcohol** n	
1 ≤ glass/week	76
2-7 glasses/week	20
>5 glasses/occasion	3
missing	1
**NYHA class** n	
II	12
III	80
IV	8
**CCI** mean (SD)	4.0 (2.2)
**Blood pressure** mean (SD)	
Systolic	121.3 (22.9)
Diastolic	67.3 (11.1)
**Pulse** mean (SD)	75.0 (11.6)
**Pulse rhythm** n	
regular	39
irregular	51
missing	10
**Medication** n	
ACEI/ARB	72
β-blockers	87
MRA	49
Diuretics	95

**Key for abbreviations**: NYHA class = New York Heart Association Functional.

Classification, CCI = Charlson Co-morbidity Index, ACEI = Angiotensin-Converting Enzyme Inhibitors, ARB = Angiotensin Receptor Blocker, β-Blockers = Beta blockers, MRA = mineralocorticoid receptor antagonists.

At baseline, patients scored a mean level of 20.7 ± 6.6 for self-care behavior (possible range 9–45), and 12.2 ± 2.0 for knowledge of HF (possible range 0–15). Regarding symptoms of depression, patients scored a mean level of 9.5 ± 6.0 (possible range 0–27).

### Patient participation over time

At baseline, patient needs had the highest scores with 23.6 ± 3.7 (84% of the max score), followed by patient involvement with 19.5 ± 3.4 (81% of the max score), and overall satisfaction with involvement in care with 7.8 ± 1.8 (78% of the max score). The lowest score was for information with 14.8 ± 3.4 (74% of the max score) (Table 2). The mean score of the information scale changed significantly over time, with an increase from 14.8 ± 3.4 at baseline to 16.2 ± 3.0 at the 12-month follow-up, indicating higher involvement over time (p = 0.003). The post hoc analysis showed that participation in terms of information increased significantly from baseline to six and twelve months.

**Table 2:** **Changes in patient participation over time based on a repeated measure ANOVA**

**Variable**	**Time**						
	**Baseline (TI)**	**1 month (T2)**	**6 month (T3)**	**12 month (T4)**	**F (df)**	**Main effect over time**	**Post-hoc** ^**a**^
	**Mean (SD)**	**Mean (SD)**	**Mean (SD)**	**Mean (SD)**		**P-value**	
Patient involvement (n = 47)	19.53 (3.44)	19.28 (3.27)	19.43 (3.04)	20.21 (3.26)	1.997 (3)	0.117	
Information (n = 47)	14.77 (3.43)	15.96 (2.94)	16.23 (3.29)	16.15 (2.98)	4.861 (3)	0.003	B p = 0.009 C p = 0.008
Patient needs (n = 48)	23.60 (3.72)	24.54 (3.31)	24.60 (3.30)	24.54 (3.31)	1.998 (2.6)^b^	0.126^b^	
Overall satisfaction involvement (n = 49)	7.82 (1.80)	7.96 (1.64)	7.84 (1.88)	7.92 (1.64)	0.153 (3)	0.928	

Possible score range (a higher score indicates more positive ratings): Patient involvement 6–24; Information 5–20; Patient needs 7–28; Overall satisfaction involvement 1–10.

^a^= Bonferroni corrected p-values. Significant differences are reported as A = T_1_-T_2_, B = T_1_-T_3_, C = T_1_-T_4_, D = T_2_-T_3_, E = T_2_-T_4_, F = T_3_-T_4_.

^b^= Huynh-Feldt correction according to violation of the assumption of sphericity.

### Factors associated with participation in care

The bivariate correlation analysis showed that lower degree of symptoms of depression, better self-care, higher levels of knowledge about HF, male sex, lower age, cohabitation and having home help services were associated with a higher degree of participation, measured by one or more of the four scales (p < 0.10). The scales measuring different aspects of participation correlated significantly with each other (r = 0.22-0.62, p < 0.05) (Table 3).

**Table 3:** **Bivariate associations for different factors correlated with the four scales measuring aspects of patient participation at baseline**

**Items**	**Patient involvement**	**Information**	**Patient needs**	**Overall satisfaction involvement**
Patient involvement				
Information	0.35**			
Patient needs	0.38***	0.62***		
Overall satisfaction involvement	0.22*	0.32**	0.49***	
Symptoms of depression	−0.04	−0.13	−0.32**	−0.34***
Self-care	−0.38***	−0.44***	−0.41***	−0.29**
Knowledge	0.19 †	0.20*	0.23*	0.11
Co-morbidity	−0.01	−0.01	0.03	0.12
NYHA	0.19†	0.08	−0.06	−0.20†
Sex	−0.13	−0.19†	−0.17†	−0.10
Age	−0.30**	−0.10	−0.01	−0.02
Cohabitation	0.33**	−0.04	0.10	0.19†
Housing	−0.15	−0.04	0.06	−0.08
Home-help	−0.23*	0.15	−0.01	−0.06
Education	0.06	0.02	0.07	−0.01

*p < 0.05, **p < 0.01, ***p < 0.001.

†p < 0.10.

NYHA = New York Heart Association Functional Classification.

The regression analysis showed that better self-care behavior, living together with someone and younger age were significantly associated with higher-rated importance of involvement (F(3,92) = 13.13,p < 0.001). These variables explained 30% of the total variance in this scale (Table 4). The degree to which patients stated that they had received information about HF was significantly associated with better self-care behavior, better knowledge about HF, male sex and having home help services (F(4.91) = 9.96, p < 0.001). These variables explained 30% of the total variance in the information scale. Lower degree of symptoms of depression, better self-care behavior and better knowledge about HF was significantly associated with how patients needs were fulfilled with regard to asking questions and being treated with respect by health care professionals (F(3,94) = 12.14, p < 0.001). These variables explained 28% of the total variance in this scale. Overall satisfaction with involvement in HF care was significantly associated with lower degree of symptoms of depression, better self-care behavior and living together with someone (F(4,93) = 7.13, p < 0.001). NYHA class contributed to the overall fit of the model, although this was not significantly associated with satisfaction of involvement (p = 0.085). These variables explained 24% of the total variance of the overall satisfaction with involvement in HF care.

**Table 4:** **Factors associated with participation at baseline, based on stepwise regression with backward elimination**

**Outcome variable**	**Predictor variable**	**B (SE)** ^**a**^	**95 % CI for B**	**P-value**
Patient involvement (n = 96)	Self-care	−0.20 (0.05)	−0.30, −0.11	<0.001
	Cohabiting	2.17 (0.68)	0.82, 3.52	0.002
	Age	−0.08 (0.04)	−0.16, −0.01	0.032
	Model statistics F(3, 92) = 13.13, p < 0.001, R^2^ = 0.30			
Information (n = 96)	Self-care	−0.24 (0.05)	−0.33, −0.14	<0.001
	Knowledge	0.39 (0.16)	0.08, 0.70	0.014
	Female sex	−1.52 (0.67)	−2.85, −0.18	0.026
	Having home help	1.61 (0.65)	0.32, 2.90	0.015
	Model statistics F(4, 91) = 9.96, p < 0.001, R^2^ = 0.30			
Patient needs (n = 98)	Symptoms depression	−0.15 (0.06)	−0.27, −0.03	0.015
	Self-care	−0.22 (0.05)	−0.33, −0.12	<0.001
	Knowledge	0.48 (0.17)	0.14, 0.82	0.007
	Model statistics F (3, 94) = 12.14, p < 0.001, R^2^ = 0.28			
Overall satisfaction	Symptoms depression	−0.09 (0.03)	−0.15, −0.02	0.009
involvement (n = 98)	Self-care	−0.08 (0.03)	−0.14, −0.03	0.003
	Cohabitation	1.06 (0.37)	0.32, 1.79	0.005
	NYHA III-IV	−1.01(0.58)	−2.17, 0.14	0.085
	Model statistics F(4,93) = 7.13, p<0.001, R2 = 0.24			

Key for abbreviation: NYHA class = New York Heart Association Functional Classification.

Self-care; lower numbers indicate better self-care, Knowledge; higher numbers indicate better knowledge, Symptoms of depression; lower numbers indicate less symptoms of depression, Age; lower numbers indicate younger age, Dichotomous variables: Sex (men = 0, women = 1), Cohabitation (No =0, Yes = 1), Home help (No =0, Yes = 1), NYHA (NYHA class II = 0, NYHA class III-IV = 1).

^a^Unstandardized regression coefficient.

## Discussion

This study is the first to explore how patients with HF view participation in structured home care. Our main findings were that although the patients experienced a fairly high level of satisfaction with participation in care at baseline, there was a significant improvement over time for participation regarding received information after receiving structured home care. This was encouraging since the structured home care focused on patient education. Furthermore, this is the first study to show that higher-rated aspects of patient participation are consistently associated with better self-care behavior in HF.

### Different aspects of patient participation and change over time

We had expected a significant improvement of patient participation with regard to patient involvement, information, patient needs and overall satisfaction with involvement after receiving structured home care. Structured home care had multiple objectives aiming to facilitate the patient’s care. The team members had received education about HF and treatment, including the importance of giving structured and individualized information to increase patients’ knowledge and self-care. The education did not explicitly focus on how patient participation could be strengthened, and perhaps this is reflected in the result in the present study. However, the aspect of participation with regard to received information increased significantly from baseline to six and twelve months. Patients had received information about their condition, why and how examinations were done and what could happen if their HF deteriorated. This was a significant improvement when receiving structured home care. Findings from qualitative studies with patients suffering from HF emphasize the importance of an exchange of care-related information to enable participation in care [16,21]. Furthermore, being well-informed contributes to patients’ perceived participation [1,16], which is consistent with the results related to received information in this study.

A crucial question that remains unanswered is whether the patients already participated sufficiently in the care. We have no cut-off values in the scales measuring participation, and it is therefore difficult to stipulate what is a good and sufficient outcome for participation. In the Swedish National Patient Survey, participation in relation to care and treatment was scored between 76-81% within different types of care settings [53]. These results are similar to how patients with HF estimated satisfaction with overall involvement in the present study. Compared to patients hospitalized due to MI [37], the overall satisfaction with involvement was scored lower among patients with HF; 78% of the maximum score compared to patients with MI who scored 84%. Based on that, the overall satisfaction with involvement in HF home care may have some potential for improvements. Nevertheless, these comparisons between patient groups must be made with caution as they may have been influenced by the patients’ characteristics, severity of illness, co-morbidity, age and also the type of care given.

Another question is whether we can expect an increase or change over time in all aspects of participation. For instance, the aspect of patients’ definition of the importance of involvement could hypothetically be more stable. Hence, Say and colleagues reported in a review how preferences for participation could change, but there was no clear pattern regarding patients’ willingness to participate in decision-making due to the illness experience. In some studies, increased illness experience was associated with increased willingness to participate, while other studies showed the exact opposite results [39], thus illustrating the complexity of drawing conclusions in connection with patients’ preferences for participation.

### Factors associated with patient participation

There was a consistent association between the participation scale and self-care behavior. Different aspects of patient participation, such as higher scoring for the definition of the importance of involvement, receiving information, fulfilled needs and higher overall satisfaction with involvement in care were all significantly associated with self-care behavior.

Patient participation includes different aspects, such as making decisions [21], and managing one’s condition [20]. Decision-making is an underlying process in self-care, where the ability to reflect is of importance for sufficient self-care decisions [4]. Our results showed significant associations between self-care and patient participation. However, based on our results, we cannot exclude that it is participation that affects self-care. Most likely, it is a reciprocal association between self-care and participation.

Knowledge can be seen as a prerequisite to be able to participate in care [15,54]. Better knowledge of the HF condition and its symptoms and treatment were associated with higher ratings for received information and fulfilled needs, two of the aspects of patient participation. This confirms earlier findings showing that knowledge gained by obtaining and understanding information has an important role for participation [1,16]. Eldh and colleagues reported that patients expressed that having knowledge, which was beyond merely receiving information, was described as participation and led to feelings of being able to manage the situation [16]. From this we can assume that when patients had received information about their condition and also had the opportunity to ask questions related to this information, their knowledge about HF may have been influenced. However, knowledge is a foundation for successful HF self-care [31], but despite increased knowledge, outcomes do not always improve [55]. It may be important to pay attention to patient participation, as we have found a significant association between different aspects of participation and self-care.

From earlier studies it is known that depressive symptoms influence patients’ perception of health, their overall life situation [56], and adherence to self-care [57]. Tambuyzer and colleagues described a relationship between patient involvement and patient satisfaction, where involvement predicted patient satisfaction [58]. We also found that a lower degree of depressive symptoms was associated with higher ratings for fulfilled needs and overall satisfaction with involvement in care.

### Limitations

This study has limitations that need to be acknowledged. It was based on a small consecutive sample, and there may also be a selection bias due to the large number of patients who declined to participate. This may affect the generalizability of the results. Overall, the group of patients who received home care for their HF was severely ill and frail. However, it is not uncommon that a high percentage of severely ill patients with HF decline to study participation; this was recently reported by Zambrosky and colleagues [59]. Another limitation was the large number of dropouts between baseline and the 12-month follow-up assessment (n = 51). This was partly expected as the sample was in need of home care. To check if our non-significant findings in the ANOVA were the result of lack of statistical power, a post hoc power analysis was conducted using G*Power 3.17. We made a calculation based on a small and a medium effect size for a repeated measure ANOVA, defined by Cohen (f = 0.10 vs. f = 0.25) [60,61]. The other parameters for the calculation were a sample size of 47 individuals, α = 0.05, and four measurement points. From this calculation we identified a power problem in detecting a small effect (1-B = 0.33), while the power was sufficiently large to detect a medium effect (1-B = 0.99). In addition, the final sample and the dropout did not differ regarding sex and age.

Furthermore, the patients had a mean age of 82 years, which is older than the mean age of the Swedish HF population [62]. The majority of the patients also had severely symptomatic HF classified in NYHA-class III (80%) or IV (8%), with a relatively high burden of co-morbidities. All these circumstances must be taken into consideration when interpreting the results and also affects the generalizability of the results to the whole group of patients diagnosed with HF. With this study design we have examined associations between patient participation and other factors, but this design cannot explain causal relationships. To be able to comment on this, further studies are required. Investigating factors that may mediate or moderate this association could also contribute to a better understanding of the relationship between self-care and participation. This could be done by examining the predictors for change in participation over time, but the sample at the one-year follow-up in the present study was too small for this analysis.

The implementation of *The heart failure at home model* could also be criticized for being weak as there was only one educational day to introduce the model. This day included education in heart failure and self-care. In self-care, the patient’s involvement is a key factor, which was an important part of the training. In addition, the centers were audited to ensure that the home care model was implemented throughout the study. We realize that we would have needed an RCT to state that this model with structured home care was better than standard homecare, and we can therefore only comment on change with regard to structured home care. However, the study adds to the current knowledge since we particularly studied the HF patients’ participation in their care and factors associated with patient participation. This information is valuable when designing future interventions for these severely ill and vulnerable patients.

The measurement properties of the majority of the instruments used in this study had previously been validated. For the present study we also wanted to evaluate the internal consistency of these scales in our sample. However, the Swedish translation of the Dutch Heart Failure Knowledge Scale [47] has not previously been tested for validity and reliability in a Swedish sample. To test internal consistency we used KR-20 rather than Cronbach’s alpha according to the dichotomous response format. In congruence with the original scale, the Swedish version demonstrated a low KR-20, which could be a limitation. Nonetheless, in some type of instruments all items are not expected to be related to each other. Instead, the items define the construct rather than being defined by it. When this is the case, it may not be relevant to use tests that are based on homogeneity [63], which could be a possible explanation for the low Cronbach’s α and KR-20 values related to the knowledge instrument. Another limitation may be that we treated the scales as continuous variables in the regression and ANOVA analyses. The reason was that there is no non-parametric alternative to multiple regression analysis, and therefore we have chosen to take this route during the analyses. The reason why Spearman’s correlations were used was that several of the predictor variables were ranked only as categories, i.e. NYHA and educational levels. However, there are divergent views on how this kind of scales should be handled in the analysis stage [64].

## Conclusion

We found that elderly, severely ill patients with HF who had a high prevalence of co-morbidities experienced a fairly high level of satisfaction with participation in structured home care. Patient participation has previously been rarely assessed in patients with HF. Our main findings show that there was a significant improvement over time for participation regarding received information when receiving structured home care. Furthermore, patient participation was consistently associated with self-care behavior. These results need to be investigated further, which may be interesting to target in future interventions aiming at improving self-care.

## Abbreviations

HCP: Health care professionals
HF: Heart failure
MI: Myocardial infarction
NYHA: New York Heart Association
